# Visible Human Project^®^ female surface based computational phantom (Nelly) for radio-frequency safety evaluation in MRI coils

**DOI:** 10.1371/journal.pone.0260922

**Published:** 2021-12-10

**Authors:** Gregory M. Noetscher, Peter Serano, William A. Wartman, Kyoko Fujimoto, Sergey N. Makarov

**Affiliations:** 1 Department of Electrical and Computer Engineering, Worcester Polytechnic Institute, Worcester, Massachusetts, United States of America; 2 NEVA Electromagnetics, LLC, Yarmouth Port, Massachusetts, United States of America; 3 Ansys, Inc., Canonsburg, Pennsylvania, United States of America; 4 Athinoula A. Martinos Center for Biomedical Imaging, Massachusetts General Hospital, Charlestown, Massachusetts, United States of America; 5 Center for Devices and Radiological Health, US Food and Drug Administration, Silver Spring, Maryland, United States of America; Information Technology University, PAKISTAN

## Abstract

Quantitative modeling of specific absorption rate and temperature rise within the human body during 1.5 T and 3 T MRI scans is of clinical significance to ensure patient safety. This work presents justification, via validation and comparison, of the potential use of the Visible Human Project (VHP) derived Computer Aided Design (CAD) female full body computational human model for non-clinical assessment of female patients of age 50–65 years with a BMI of 30–36 during 1.5 T and 3 T based MRI procedures. The initial segmentation validation and four different application examples have been identified and used to compare to numerical simulation results obtained using VHP Female computational human model under the same or similar conditions. The first application example provides a simulation-to-simulation validation while the latter three application examples compare with measured experimental data. Given the same or similar coil settings, the computational human model generates meaningful results for SAR, B1 field, and temperature rise when used in conjunction with the 1.5 T birdcage MRI coils or at higher frequencies corresponding to 3 T MRI. Notably, the deviation in temperature rise from experiment did not exceed 2.75° C for three different heating scenarios considered in the study with relative deviations of 10%, 25%, and 20%. This study provides a reasonably systematic validation and comparison of the VHP-Female CAD v.3.0–5.0 surface-based computational human model starting with the segmentation validation and following four different application examples.

## Introduction

Quantitative assessment of radio frequency (RF) absorption experienced by a patient undergoing a Magnetic Resonance Imaging (MRI) procedure is prohibitively difficult to obtain due to the invasive nature of such measurements. Numerical simulations with computational human models are used to estimate electric field strength, specific absorption rate (*SAR*), current densities in and around tissues, and expected temperature rise [1–9] and to comply with relevant international and domestic standards [10–14]. While well over 30 whole-body human models created from patients of various age, body composition, sex and race exist, (cf. for example, [15]), documented validation of these models (which involves verification against independent measurements and simulations under the same or similar settings) is not always available.

**Fig 1:**
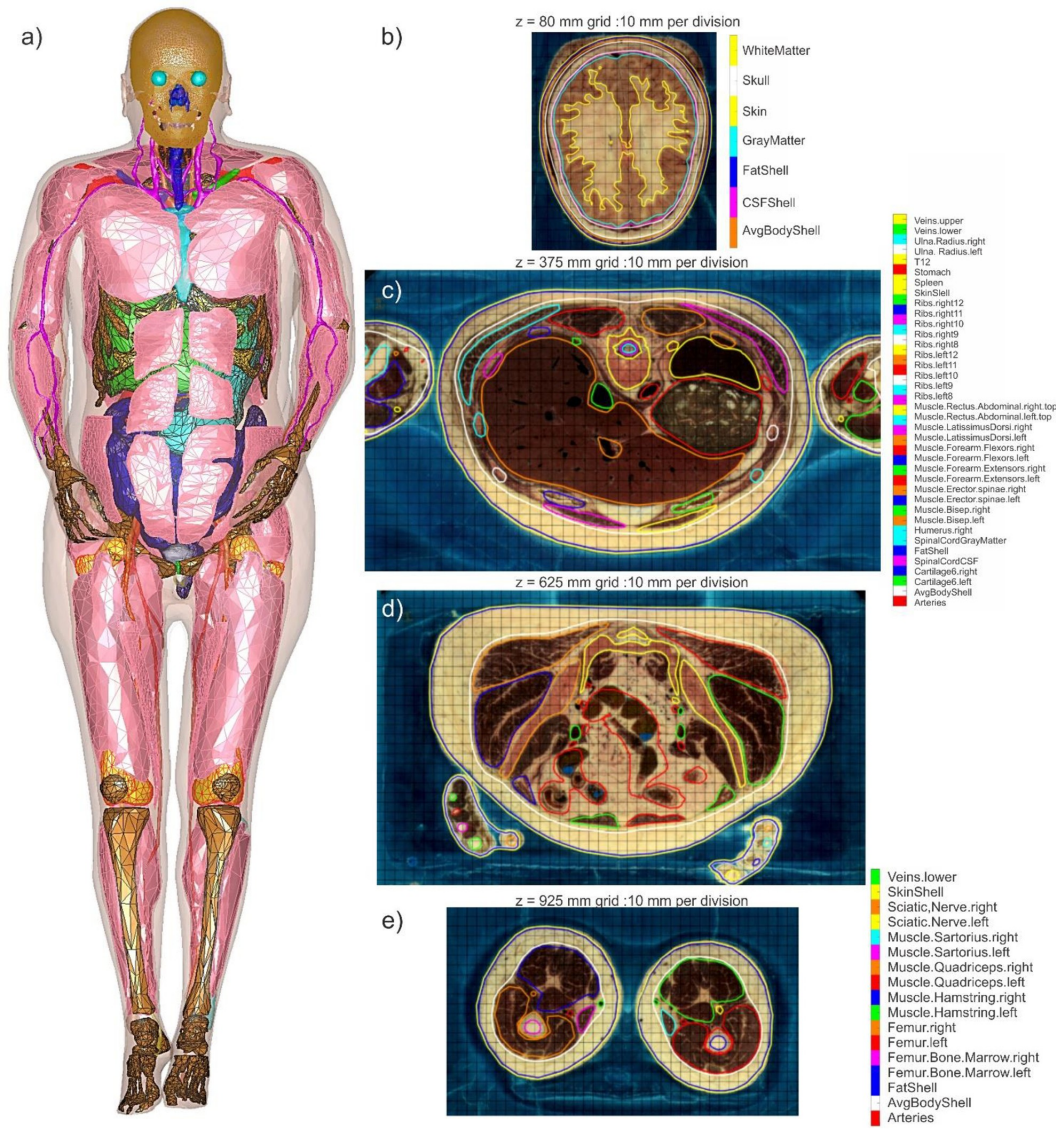
a)–Full-body Visible Human Project Female v5.0 CAD based computational phantom. The phantom is composed of 249 individual structures. Some individual muscles are removed for clarity. b-e) Examples of coregistration maps for surface meshes in different transverse planes with tissue labeling. In case b), non-anatomical separation between scalp and skull was corrected. In case d), the tissue labeling list is not shown. The complete full-body coregistration maps with the vertical resolution of 1 mm and with tissue labeling in every cross-sectional plane are available online in *.mp4 format [24] for independent inspection and verification purposes.

The Visible Human Project (VHP) Female v.3.0–5.0 computational human model (also known under the nickname ‘Nelly’ to users of Dassault Systèmes SIMULIA software CST), shown at left in Fig 1, is an anatomically accurate heterogeneous female (~60 years old, ~ 88 kg, BMI of ~36, obese, with heart pathology) surface-based human body model. It was constructed from the photographic cryosection data of the VHP conducted by the US National Library of Medicine. Its construction has been well documented in the literature [15–21], but several points are worth emphasizing here:

1. There are 249 distinct components or triangular 2-manifold surface meshes (with an additional 40 characterizing embedded implants). No intersections or joint faces between discrete meshes are allowed, enabling unique assignment of electromagnetic, thermal, or other material properties.
2. The source data for the model are freely and publicly available [22,23]. The complete co-registration data for all model cross-sections are also made publicly available [24].

One advantage of the model is its topological and computational simplicity. The model size is purposely limited to approximately 0.4 M triangular facets in total with an attempt to keep the anatomical accuracy within the body within 2–7 mm. This makes it possible to apply virtually any commercial or custom-tailored finite-element or boundary element computational solver in a very reasonable amount of time. Nearly identical results are obtained when using different software packages and numerical methods [25].

Yet another advantage is the separation of the muscular system of the body into individual muscle objects (about 50 in total).

Some recent uses of the VHP Female v3.0–5.0 computational human model have been documented in the literature–cf. for example [25–42]–and compared with experimental data on radio frequency propagation. A number of studies have focused on the characteristics and behaviors of antennas near the human body [26–34]. Others have examined through simulation various biomedical applications including transmission channel modeling [35], transcranial direct current stimulation [36], estimation of bone density [37], gastroenterology [38,39], *SAR* simulations in MRI coils at 3 T [40], and safety of active implants under MRI procedures [41,42]. A simplified version of the model, VHP-Female v. 2.2, has over 500 registered users and is available for download online [43].

However, a systematic model validation and comparison have not been performed. The present study attempts to fulfill this task using published experimental [44–49] and modeling [72] data, and starting with the initial segmentation validation. The most relevant context of use is a non-clinical assessment model for female patients of age 50–70 years with a BMI of 30–36 during 1.5 T and 3 T based MRI procedures. Note that, compared to validation and comparison methodologies for models based on patients that are still living and available for direct field measurements [50], the present study has to employ different human subjects with the exception of the simulation-to-simulation validation in the first application example.

## Materials and methods

### Validation of model segmentation

The co-registration method has been used, which implies direct superposition of transverse cross-sections of all surface tissue meshes onto the original cryosection images. An in-house MATLAB module (Fig 1B–1E) was written that performs such superposition with the resolution of 1 mm and simultaneously labels all tissue meshes which are present for a given cross-section. Its output is a scanning sequence in *.mp4 format [24].

Further visual assessment was performed by a number of anatomical experts in their respective areas with the participation of Profs A. Nazarian (orthopaedics), A. R. Opotowsky, (cardiovascular systems), V. Poylin, (gastroenterology), E. K. Rodriguez (nusculoskeletal tissue components), A. Pascual-Leone (cranial and intracranial anatomy) from Beth Israel Deaconess Med. Center, and Massachusetts General Hospital, Boston MA, and Prof. G. Haleblian (urology) from Saint Vincent Hospital, Worcester MA. The results will be summarized in the results section.

### Numerical simulation method used in application examples

In each reported case presented herein, Ansys Electronics Desktop (HFSS) 2019 R1 finite-element solver with adaptive mesh refinement has been employed to solve Maxwell’s equations in three-dimensional space. In this way, the model is considered as an arbitrary (inhomogeneous) isotropic medium with piecewise-constant electric permittivity *ε* having the units of F/m and with constant magnetic permeability *μ* having the units of H/m. After Maxwell’s equations are solved for an electric field (or the electric field intensity) ***E***(***r***,*t*) [V/m] and for a magnetic field (or the magnetic field intensity) ***H***(***r***,*t*) [A/m], volumetric electric current is directly related to the electric field by a local form of Ohm’s law,

J(r,t)=σ(r)E(r,t) (1)

where *σ*(***r***) is (generally piece-wise constant) medium conductivity with the units of S/m, ***r*** is the position vector (or location in 3D space) and t is time.

In validation examples 2 and 4, the solutions of these simulations have been coupled to Ansys Mechanical 2019 R1 via Ansys Workbench. Pennes’ bioheat equation, based on the heat diffusion equation, is a standard approximation for heat transfer in biological tissues [51–53] partially implemented in the Ansys Mechanical software. It has the form

$$\rho (r)C(r)\frac{\partial T}{\partial t}(r,t)=\nabla \cdot (k(r)\nabla T(r,t))=\rho (r)Q(r)+\rho (r)SAR(r)-\rho_{b}C_{b}B(r)(T(r,t)-T_{b})=0$$ (2)

where *T*(***r***,*t*) is the local temperature, *ρ*(***r***) is density, *C*(***r***) is specific heat capacity (at constant pressure), *Q*(***r***) is the metabolic heat generation rate, *B*(***r***) is the perfusion rate [1/s]; index *b* is related to blood.

All electromagnetic, mechanical, and thermal tissue properties used in the present study are catalogued in the IT’IS database [54]. The electromagnetic material properties are given as a function of frequency, which is suitable for the present analysis.

The local *SAR* (W/kg) is defined through averaging the dissipated power per unit mass over a small (ideally infinitesimally small) volume *V*, that is

$$SAR(r)=\frac{1}{V}\int_{V}\frac{\sigma (r)}{2\rho (r)}{\left|E(r)\right|}^{2}dV$$ (3A)

Here, |***E***(***r***)| is the electric field magnitude at the observation point. The body-averaged or the whole-body *SAR*_*body*_ is given by averaging over the entire body volume, as

$${SAR}_{body}=\frac{1}{V_{body}}\int_{V_{body}}\frac{\sigma (r)}{2\rho (r)}{\left|E(r)\right|}^{2}dV$$ (3B)

Similarly, *SAR*_1*g*_ is given by averaging over a cubic volume with a mass of 1 g

$${SAR}_{1g}(r)=\frac{1}{V_{1g}}\int_{V_{1g}}\frac{\sigma (r)}{2\rho (r)}{\left|E(r)\right|}^{2}dV$$ (3C)

*SAR*_10*g*_(*r*) is found in a similar fashion.

To solve Eq (3), the electric field is required, which is difficult to measure directly during an MRI procedure. However, it is possible to obtain the $B_{1}^{+}$ field, which can be used to predict and estimate the electric field. According to [55], various $B_{1}^{+}$-mapping methods have been proposed to measure the magnitude of $B_{1}^{+}$ components (especially for the transmit $B_{1}^{+}$ component), such as

1. using multiple acquisitions with different flip angles of spins [56,57];
2. applying identical RF pulses followed by two delays of different repetition times (TRs) [58];
3. utilizing phase-sensitive means based on composite RF pulses [59] or on the Bloch–Siegert phase shift [60],
4. employing stimulated echoes acquisition mode (STEAM) in multipulse sequences [61,62] or in a single sequence followed by a tailored gradient echo train [63].

On the other hand, there is no direct measurement to quantitatively obtain the absolute phase distribution of the transmit or receive $B_{1}^{+}$ field [55].

Local *SAR* values given by Eq (3) can now be derived from the $B_{1}^{+}$ field mapping [64]. In summary, we express the electric field form Ampere’s law in frequency domain,

$$E(r)=\frac{\nabla \times H_{1}^{+}(r)}{j\omega k(r)},\;k(r)=\varepsilon (r)-j\sigma (r)/\omega$$ (4)

Once ***E***(***r***) is known from Eq (4), the local *SAR* in Eq (3),

$$SAR(r)=\frac{\sigma (r)}{2\rho (r)}{\left|E(r)\right|}^{2}$$ (5)

can be computed. Variations of this method are available [55].

### Modeling setup for validation Example 1: Normalized SAR predicted by two different modeling techniques in 1.5 T birdcage whole body MRI coil

**Fig 2:**
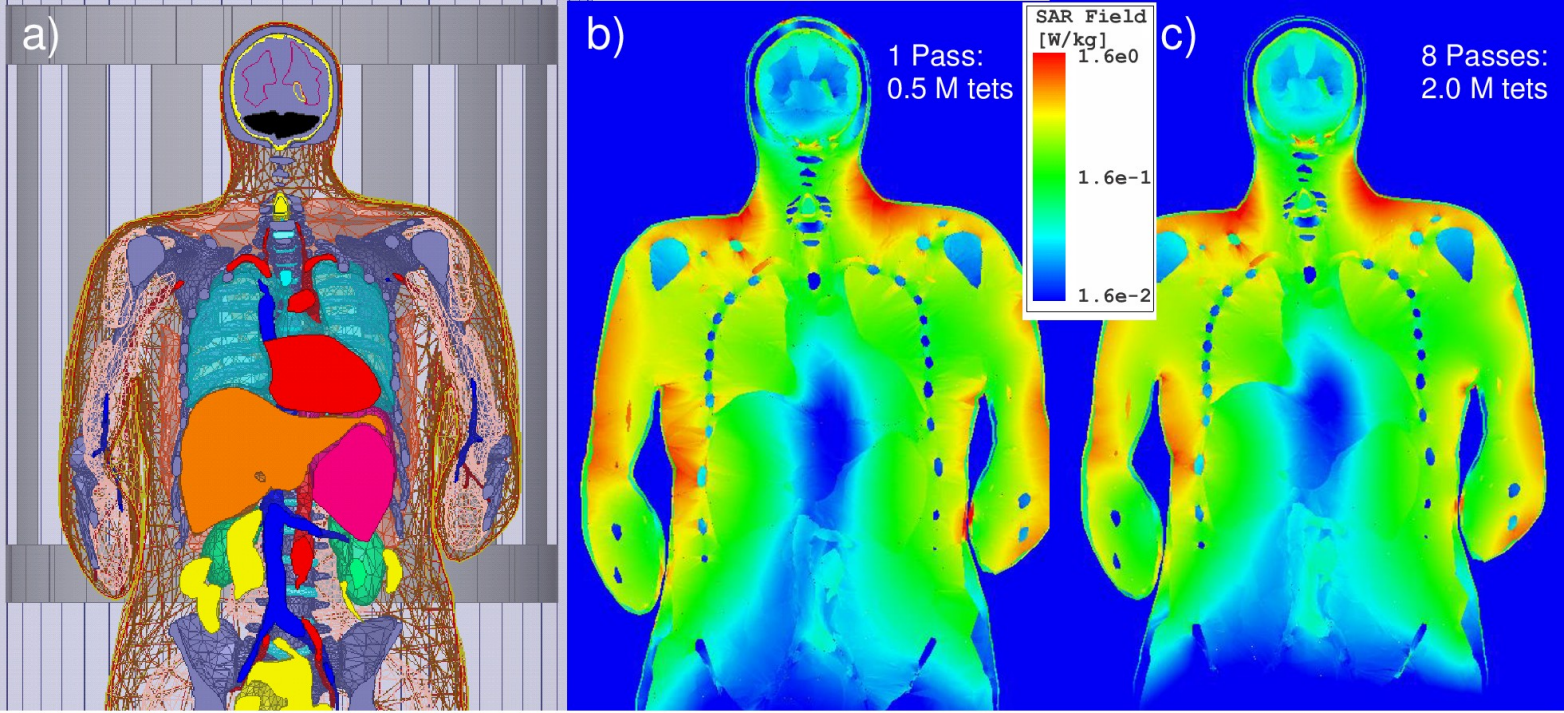
Local SAR distribution in the coronal plane for a high pass full-body RF coil operating at 64 MHz loaded with the VHP-Female v5.0 computational phantom given $B_{1}^{+}$ amplitude of 1 μT at the coil center. a)–Positioning of model within the birdcage at shoulder/heart landmark. b)–Ansys HFSS (Electronics Desktop) solution with one adaptive pass. c)–Solution with eight adaptive passes.

In the first example, a generic, whole-body high-pass birdcage coil with 16 rungs and 32 matching capacitors, loaded with the VHP Female model, is considered. The coil has a diameter of 64 cm and length of 69 cm consistent with [65]. The simulation geometry is shown in Fig 2A. Simulations have been conducted at shoulder/heart and abdominal landmarks. For the former, the coil center is oriented to coincide with the top of the T7 vertebra; the latter has the coil center located at the top of the L1 vertebra. The coil was tuned to the desired frequency of 64 MHz (*B*_0_ = 1.5 T) when loaded with the subject at each landmark as described in S1 Appendix (cf. [72]). Similar to [72], an ideal excitation was applied with 32 sources placed in the two end-rings to perform the function of the capacitors. This excitation provides results which are very similar to the conventional two-port or four-port excitations [72].

A comparison was further made with the results of Ref. [72] which was using a nearly identical high-pass birdcage coil (diameter of 63 cm and length of 70 cm), the nearly identical heart landmark, and the identical coil excitation type. However, an in-house voxel model for the same VHP-Female dataset was employed in [72] followed by the FDTD simulation method with the resolution of 5 mm.

To compare the solution variation as a function of FEM mesh density and the solution convergence trends, solutions were generated first with 1 and then with 8 adaptive mesh refinement passes, created approximately 0.5 M and 2.0 M tetrahedra, respectively. As an example, Fig 2 shows the corresponding local *SAR* distributions at two different FEM resolutions in the coronal plane for the coil loaded with the VHP-Female v3.0 computational human model given $B_{1}^{+}$ amplitude of 1 μT at the coil center. Fields solutions from Ref. [72] were also normalized given the desired magnitude of $B_{1}^{+}$ at the coil center of 1 μT. The normalization is done in the form

$$SAR\to \frac{SAR}{{{(B}_{1}^{+}/1\mu T)}^{2}}$$ (6)

The local *SAR* was computed in Ansys HFSS and then exported to MATLAB over a uniform 3D grid of 2 mm in size. Whole-body *SAR*_*body*_ was computed from this data directly in MATLAB. *SAR*_1*g*_ was also calculated by finding a volume *V*_1*g*_ surrounding the observation point having the mass of exactly 1 g, and then performing averaging according to Eq (3C). This averaging volume contains approximately 5×5×5 individual voxels (2×2×2 mm each) closest to the observation point. The observation points form a 3D sub-grid spaced of 20 mm and 10 mm, respectively. SAR over any 1g was approximated as the SAR over any 1cm^3^. *SAR*_10*g*_ was computed in the same way. In this case, the averaging volume contained approximately 1250 individual voxels (2×2×2 mm each) closest to the observation point.

### Modeling setup for comparison Example 2: Metal nail implant heating in 1.5 T birdcage whole body MRI coil

ASTM phantoms have been used as the standard method for testing compliance of implants within MRI environment [73]. The phantom is not an equivalent of intricate human body structures. Therefore, we consider this example, which employs the experimental phantom data, as a comparison, not as the validation.

**Fig 3:**
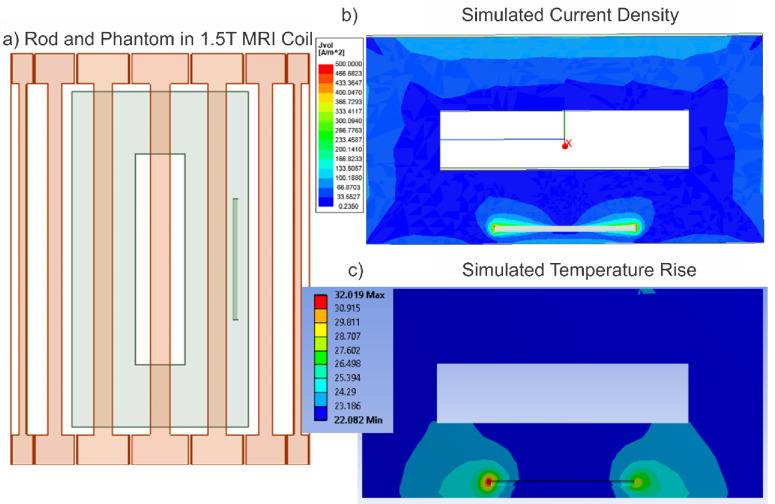
Simulations to establish calibration for experiments conducted in [43–45]. a)–The metallic nail implant placed within a homogeneous loop-like Agar phantom [43–45] at a depth of 2 cm. b)–The current density produced with a whole-phantom SAR of 4.0 W/kg. c)–The simulated temperature given a total volumetric power loss of 120 W exactly corresponding to experiment [43–45]. Simulation results produced a temperature rise of 10.02°C, slightly less than the 12.6°C experimentally observed in [43–45].

Prior to conducting simulations using the VHP Female v3.0 model, a necessary workflow and RF power calibration have been established. Fig 3 depicts to scale simulation of the original experiment performed in [44–46], which was used for calibration purposes. At left in Fig 3, a homogenous experimental AGAR gel phantom is positioned within a 1.5 T birdcage MRI coil operating at 64 MHz. All examinations [44–46] were performed with a 1.5T MR scanner (MAGNETOM Symphony, SIEMENS) with the phantom at the center of the coil. A birdcage shaped transmit/receive body coil was used there with the inner diameter of 60 cm and the length of 70 cm; these dimensions have been reproduced in the modeling setup. Coil matching and excitation was performed as described in the previous example. We again use ideal excitation which is very similar [72] to the two-port excitation used in [44–46].

A 24 cm long metallic orthopaedic nail implant of Zimmer, Inc. made of stainless steel (originally very slightly bent but modeled a straight rod of the same diameter) has been embedded in this phantom such that it is 2 cm away from the top and side edges of the phantom. Both the phantom and metallic rod have been assigned dimensions and material properties consistent with experiment [44–46].

**Fig 4:**
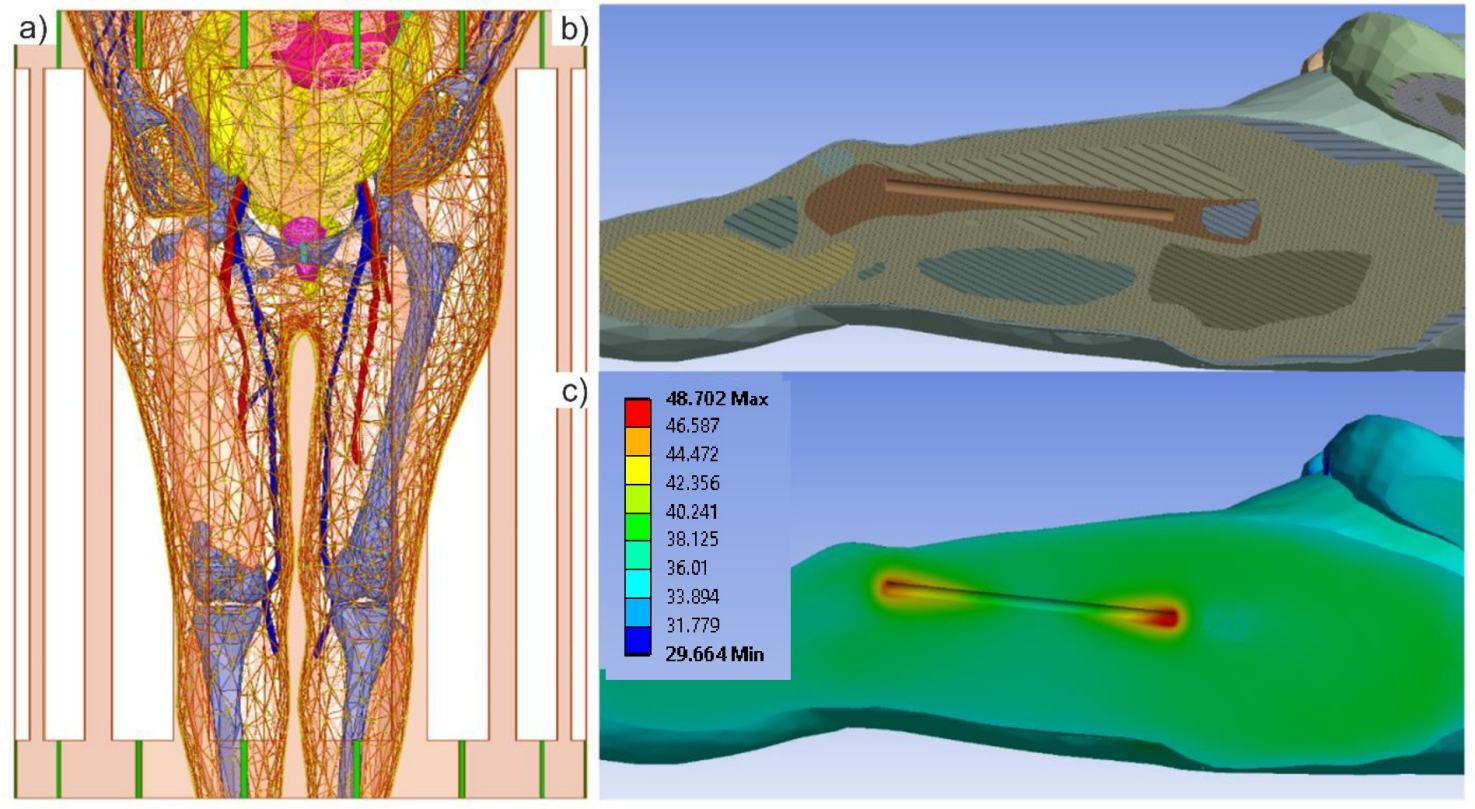
The VHP-Female computational phantom positioned with a 1.5 T MRI birdcage coil. Some body parts are removed for clarity. a)–The femur position is illustrated to show its orientation within the model–the metallic nail implant is aligned to reside within the trabecular bone structure. b)–Each individual object within Ansys Mechanical model is assigned specific thermal properties. c)–The temperature rise is shown after 900 seconds of continuous coil operation. These values correspond well with published experimental data [43–45]–see Table 2.

Fig 4 at left shows the VHP Female computational human model in a 1.5T birdcage MRI coil. The computational model has been oriented so that the center of the femur bone is aligned with the center of the coil. The left quadriceps muscle within the VHP model is not shown in Fig 4 so that the position of the femur can clearly be seen. Within the left femur, the same 24 cm long cylindrical metallic implant has been inserted. This rod is assigned material properties consistent with [44–46]. The coil is driven at 64 MHz and at a power such that the volumetric power loss within the human model is again 120 W, also consistent with the published experimental results.

A length-based mesh constraint of no edge larger than 2.5 mm was enforced for the metallic rod mesh and a total of about 636,000 tetrahedral elements were used in the Ansys HFSS simulation.

**Fig 5:**
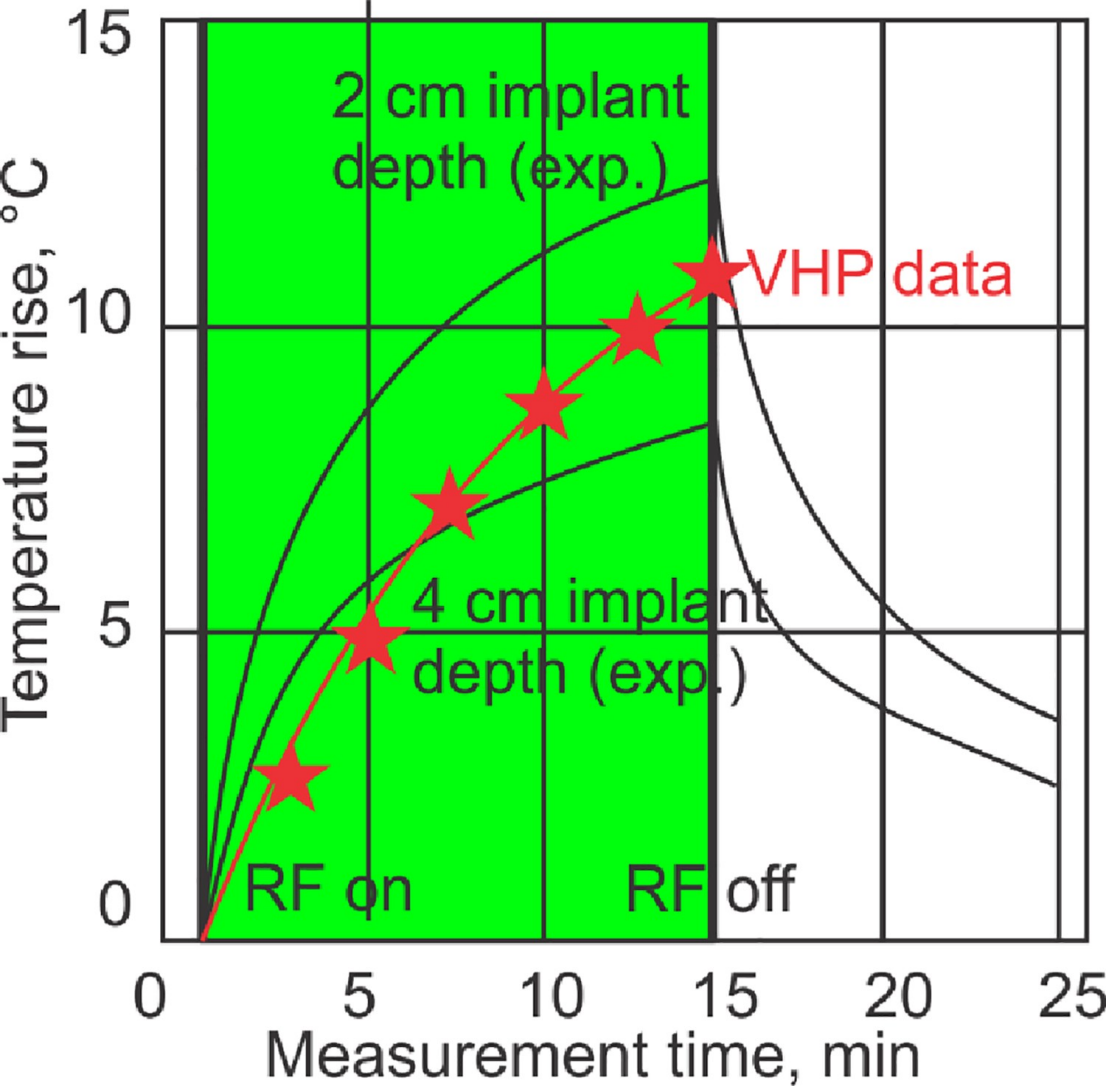
Comparison of the simulated numerical values (red stars) obtained using the VHP Female computational phantom with published experimental data (two black curves) [43–45] for the maximum temperature rise near the implant. The depth of the implant within the VHP model is *approximately* 4 cm and the maximum temperature value after 900 seconds of coil operation time is represented by the top red star. The VHP model predicts a slightly higher (by 2.75°) maximum temperature rise than what was measured in [43–45]–see Table 2. This is likely due to the different (non-homogeneous) material properties employed in the present study and the slightly angled orientation of the nail.

Once the electromagnetic simulation was complete, the results were passed to Ansys Mechanical software by linking the two simulations in Ansys Workbench. A mesh refinement was again employed on the faces of the rod to ensure that a dense enough mesh was created to capture the local temperature changes. Approximately 629,000 tetrahedral elements were used in the transient thermal simulation. Volumetric losses (*SAR*) produced by the Ansys HFSS simulation were imported into Ansys Mechanical and used as the internal heat generation source density. The RF coil and the heat sources were active for 900 s and the model was allowed to cool for another 600 s. The values for the implant temperature observed from 0 to 900 s are shown in Fig 5; they will be discussed in the Results section below.

### Modeling setup for validation example 3 $B_{1}^{+}$ maps for 3 T birdcage MRI whole body coil

The experiment [47,48] uses an 8-channel volume coil, which means each rung is independently driven. This is needed for body imaging at 3 T as the wavelength in the body is fairly short, resulting in an inhomogeneous $B_{1}^{+}$ field. This is not a birdcage design. It is a TEM coil (where each rung terminates to the shield) with a decoupling ring (not the same as a birdcage end ring). A co-simulation with a circuit solver is therefore needed to carefully tune, match and decouple all the channels (by adjusting capacitors) and then drive each channel with its own power source with the desired amplitude/phase differences.

Fortunately, in the experimental study [47], the parallel transmit body coil was driven in the so-called quadrature mode with equal current amplitude in all TX elements at 45 degrees phase differences between neighboring elements. This mimics the behavior of a birdcage resonator [47] and therefore makes it possible to use the present birdcage coil model with the nearly identical diameter of both coils (64 cm vs 63 cm [48]) and the same diameter of both RF shields, but with a somewhat longer length of our birdcage (69 cm vs ~42 cm [48]).

From experimental data for two male subjects [47], we choose healthy volunteer #2 since he has body mass of 68 kg [48] being closer to the VHP-Female human model which is important for calibration.

**Fig 6:**
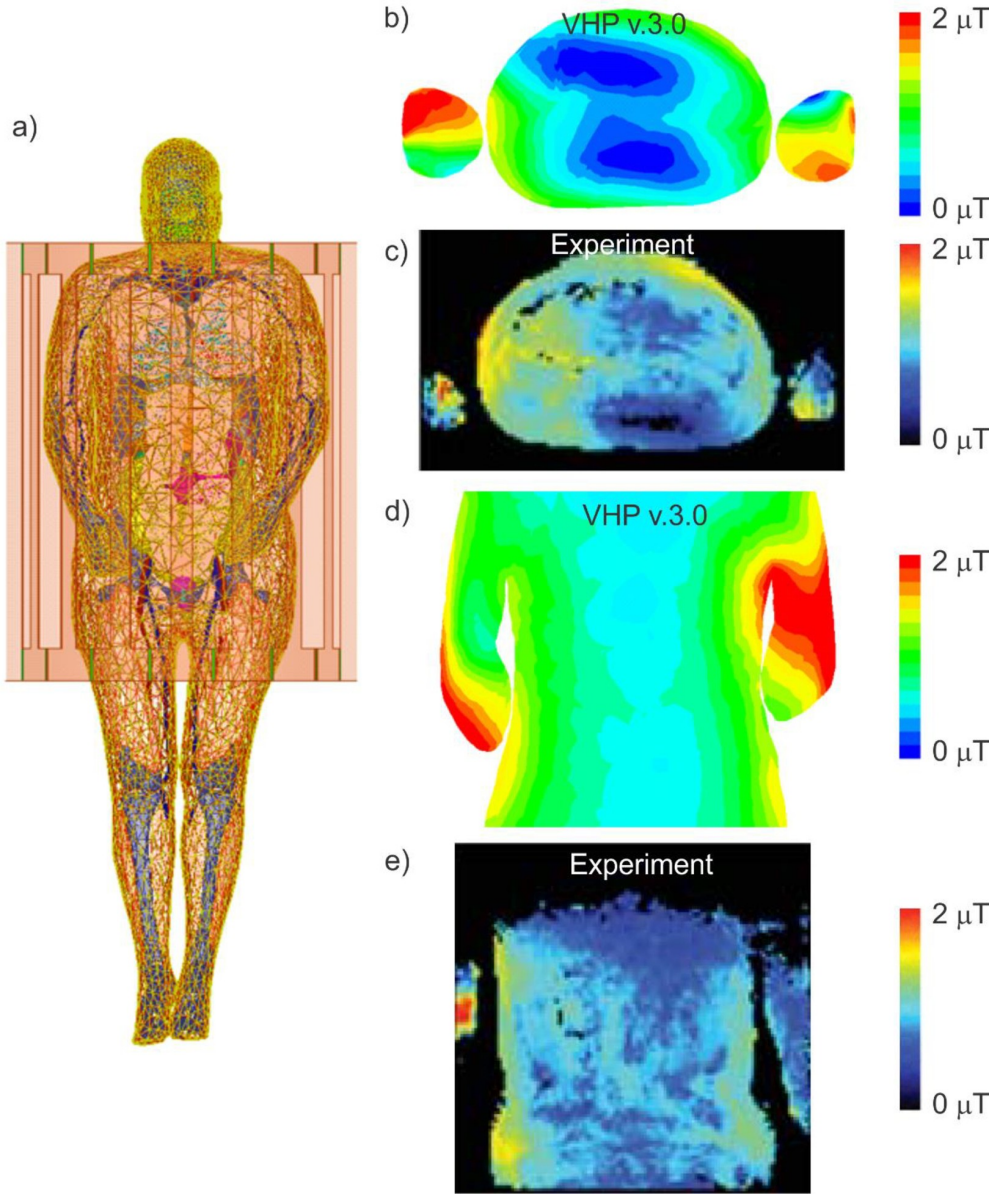
The VHP-Female computational phantom positioned with a 3T MRI birdcage coil at the abdominal landmark. a)–The model geometry is shown, providing a view of the phantom located in the coil. b)–The simulated $B_{1}^{+}$ map generated in the axial plane about the model mid-section. c)–The experimental $B_{1}^{+}$ map reported in [46,47]. d)–The simulated $B_{1}^{+}$ map generated in the coronal plane about the VHP Female model mid-section. e)–The measured $B_{1}^{+}$ map generated in a coronal plane at the approximate middle of the model. These values are also reviewed in Table 2.

The VHP Female computational human model shown in Fig 6A is then positioned in the 3 T birdcage MRI coil at the abdominal landmark to match the physical setup in [47,48]. Adaptive mesh refinement has again been employed to produce a tetrahedral mesh with about 1.7 M tetrahedra throughout the volume. Simulations have been conducted at 128 MHz (*B*_0_ = 3 T). Coil matching and excitation was performed as described in the previous examples.

We compare the $B_{1}^{+}$ distribution within the body with that obtained for healthy volunteer # 2 (male, age 29 to 43 years) [47]. The power provided to the coil has been manually calibrated to produce a whole-body *SAR* of 0.51 W/kg within the VHP Female human model to exactly comply with the experimental data [47,48].

### Modeling setup for validation example 4: Tissue heating due to single-loop coil close to skin surface at 165 MHz

**Fig 7:**
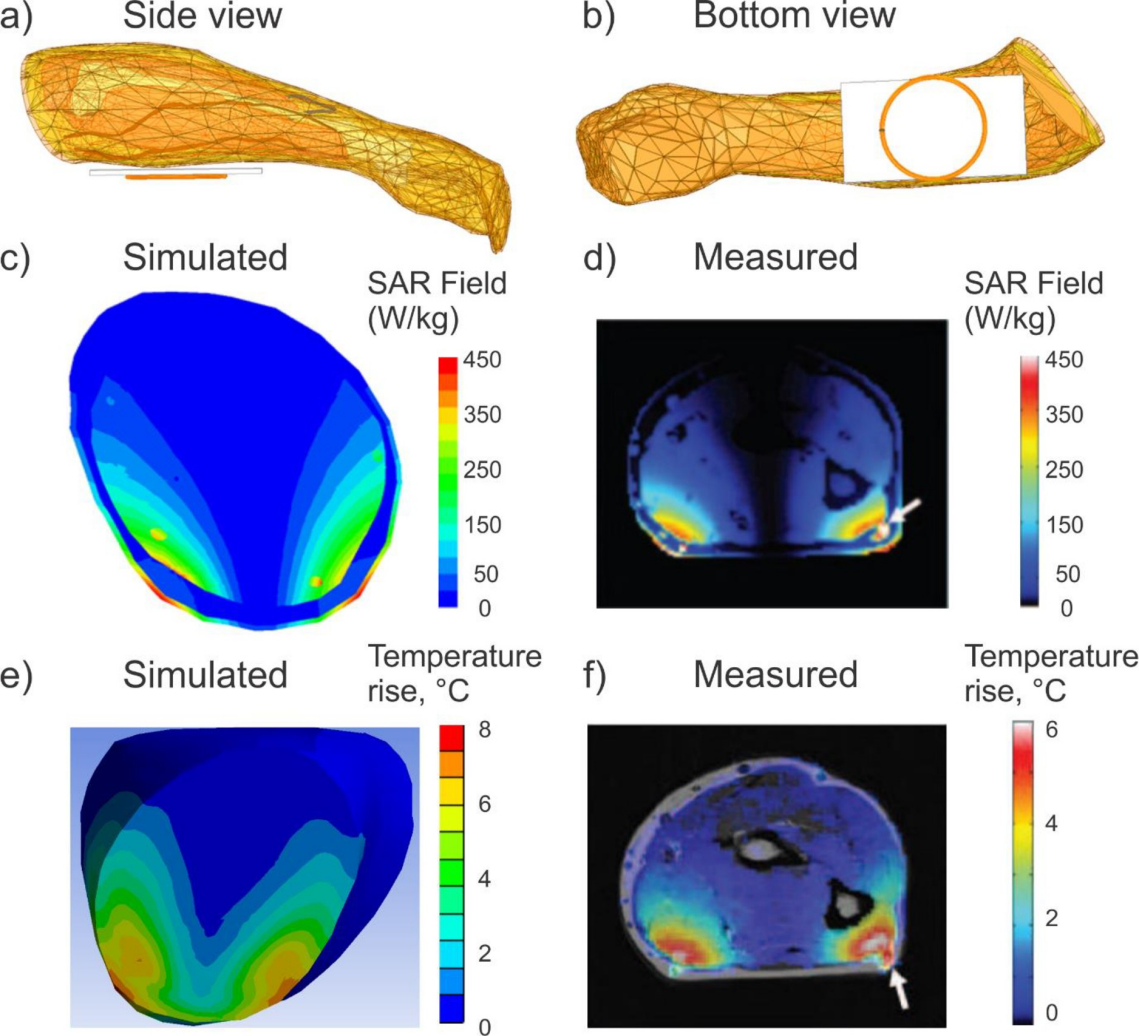
a,b)–The forearm of the VHP-Female computational human model adjacent to a single loop coil antenna operating at 165 MHz. The model geometry is shown, including internal forearm tissues, each defined with specific electromagnetic and thermal properties. c,d)–Computed (VHP-Female) and measured [49] local SAR within a plane directly above the center of the coil, respectively. Note the perfect agreement in the peak SAR. Also note the higher values generated due to the presence of the veins and nerves. e,f)–Computed (VHP-Female) and measured [49] temperature rise within a plane directly above the center of the coil, respectively, after 139 seconds. The maximum temperature difference is 1.4°C.

The final validation case models experimental setup [49]; it is shown in the top portion of Fig 7. The forearm of the VHP Female computational human model has been isolated to speed up the simulation. Internal structures, including the humerus, ulna and radius bones, extensor, flexor, triceps and biceps muscles, radial nerve, and various arteries and veins, are encapsulated within concentric layers of muscle, fat and skin tissue. The single loop antenna [49] is modeled as a 80 mm diameter copper torus with a minor diameter of 2 mm driven at 165 MHz by a 50 Ohm lumped antenna port and a thin sheet of Teflon separates the antenna from the forearm. These settings exactly correspond to experiment. All thermal and electromagnetic material properties associated with the internal body structures correspond to those supplied in [49].

In experiment [49], the forearm size and origin were not specified. However, it seen in Fig 3 of Ref. [49] that it is a male arm fully covering the coil ring. The arm of the VHP-Female model in Fig 7A and 7B is also covering the entire ring. In both models, the coil ring is underneath the arm (cf. Fig 3 of Ref. [49] and Fig 7A and 7B of the present study). However, the arm of the VHP-Female model is more round in shape since it was not supported by anything during the cryogenic freezing.

According to [49], the antenna is provided sufficient power to dissipate approximately 31 W within the forearm and produce peak local *SAR* values in the lower corners of the forearm of approximately 450 W/kg. These power conditions have been replicated in simulations with the forearm of the VHP Female model. Adaptive mesh refinement produced a volumetric mesh consisting of approximately 0.1 M tetrahedra.

Following simulation in Ansys HFSS, the geometry and resulting fields were passed via Ansys Workbench to Ansys Mechanical. Here, the human model was again re-meshed, producing just over 0.06 M elements.

The volumetric power losses (*SAR*) determined in Ansys HFSS were imported and evaluated as the heat generation source density. Heat generation sources were active for 120 s and the model then cooled via convection for 19.2 s; temperatures were recorded throughout the duration of the simulation. The corresponding temperature rise after 139 seconds in total was computed and compared with experiment.

## Results

### Segmentation validation via co-registration and anatomical expertise

After resolving multiple mesh intersections, the validation of the model segmentation shown in Fig 1A was performed by co-registration of surface meshes superimposed onto the original cryosection images (Fig 1B–1E) and correcting obvious flaws. Further visual anatomical assessment was performed by the number of experts from Beth Israel Deaconess Med. Ctr. and Massachusetts General Hospital, Boston MA. The questionable surface meshes (~12 in total, mostly bones but also soft tissues including scalp, bladder and uterus) were corrected. An example of a non-anatomical flaw is shown in Fig 1B where the scalp was non-anatomically separated from the skull during the cryogenic process, which required proper mesh adjustment. The surface deviation for all meshes was found not to exceed 2–7 mm.

The complete full-body co-registration maps with the vertical resolution of 1 mm and with tissue labeling in every cross-sectional plane are available online in *.mp4 format [24] for independent inspection and verification purposes.

### Example 1: Normalized SAR predicted by two different modeling techniques in 1.5 T birdcage whole body MRI coil

Fig 2B and 2C show the local *SAR* within the various tissues of the VHP-Female v3.0 model after 1 pass and 8 passes, respectively, for the shoulder/heart landmark. All results are normalized to the $B_{1}^{+}$ amplitude of 1 μT at the coil center. By analyzing Fig 2 we can see that

1. The accurate solution with eight adaptive mesh refinement passes generates a more realistic *SAR* distribution, especially with regard to the local *SAR* –see Table 1. In particular, two non-physical maxima of the *SAR* observed at the top of the head are no longer present.
2. The accurate solution with eight adaptive mesh refinement passes and the coarse solution with one adaptive pass generate approximately the same whole-body *SAR* and *SAR* distribution maps, but considerably different peak local *SAR* values–see Table 1. Note that the coil has been retuned separately in both cases.
3. The maximum *SAR* for the present landmark is observed in the upper shoulder/neck area and in the arms area.

**Table 1:** Comparison of *SAR* values predicted using the VHP-Female surface CAD model with the values predicted by a 5 mm voxel model derived from the identical image dataset [71]. Nearly identical high-pass birdcage coil dimensions, coil landmark, and excitation type were used. All results are normalized to 1 μT $B_{1}^{+}$ field at the coil center.

Source	Method	Model	Coil landmark	Whole-body *SAR*	Max. non-averaged local *SAR*	Max. 1g local *SAR*	Max. 10g local *SAR*
Present report	FEM—1 adaptive pass (Ansys)	CAD VHP-Fem. v. 3.0 88 kg 64 MHz	Shoulder/heart (top of vert. T7)	0.16	44.5	5.22	2.61
Present report	FEM—8 adaptive passes (Ansys)	CAD VHP-Fem. v. 3.0 88 kg 64 MHz	Shoulder/heart (top of vert. T7)	0.13	12.0	1.61	1.37
Ref. [71]	FDTD Voxel size 5 mm	Voxel Vis. Human Female 64 MHz	Heart	0.12	NA	1.78	NA

Table 1 compares the computed *SAR* values with the values obtained in Ref. [72] under nearly identical conditions. For both models and both methods, the whole body *SAR* and the maximum *SAR*_1*g*_(***r***) differ by 8% and 10%, respectively.

A comparison with a variety of other modeling reference sources [66–76] has also been made both at 1.5 T and 3 T. It was found that the *SAR* results calculated when using the VHP Female v3.0 model are strictly within the bounds of all other reported values.

### Comparison Example 2: Metal nail implant heating in 1.5 T birdcage whole body MRI coil

The computed volumetric current density within the phantom is shown at top right in Fig 3. This density matches well with the published result; it was produced by adjusting coil power to exactly replicate the RF exposure given in [44–46]–a whole-phantom *SAR* of 4.0 W/kg and a volumetric power loss density of 120 W in the phantom. The temperature simulation is shown at bottom right of Fig 3. Given the above power loss density as an internal heat generation source, the total temperature rise within the human model was 10.02°C. This value is slightly less than the 12.6°C observed in [44–46]. This small disparity may be due to differences in material properties, given that the experiments conducted in [44–46] did not explicitly provide permittivity and conductivity values, but did state that values were ‘equivalent to that of muscular tissue,’ which were replicated for the present work. However, it is close enough to give confidence in the adequate simulation setup, enabling the extension of this methodology to the case involving the VHP female human model.

Peak simulated temperature values for the implant within the human model are shown in the bottom right of Fig 4. A maximum temperature rise of approximately 11°C is observed at the very ends of the metallic implant.

Fig 5 shows temperature dynamics: it compares simulated numerical values (red stars) obtained using the VHP Female computational phantom with published experimental data (two black curves) [44–46] for the maximum temperature rise near the implant. The depth of the implant within the VHP model is *approximately* 3–4 cm and the maximum temperature value after 900 seconds of coil operation time is represented by the top red star.

The VHP model predicts a slightly higher (by 2.75°) maximum temperature rise than what was measured in [44–46]–see Table 2. This is likely due to the different (non-homogeneous) material properties employed in the present study and the slightly angled orientation of the nail. In the VHP Female model, the rod is aligned with the femur and represents a more realistic position that would be encountered in a clinical setting.

**Table 2:** Comparison of simulated numerical results using the VHP Female computational model with experimental results for examples 2, 3, and 4.

Validation/Comparison Example	Reference(s)	Quantity	Simulation results of this study	Published experimental data
**2** 1.5 T Full body, orthopaedic nail implant	[43–45]	Maximum Temperature Increase after 900 Seconds	~11°C Observed at implant ends (3–4 cm depth) in VHP-Female model–see Fig 5.	~8.25°C measured for Gel Phantom (4 cm depth) for the same implant–see Fig 3
**3** 3 T Full body	[46,47]	B1+ Field Maps in Body Planes	Between 0–2 μT depending on body location–see Fig 3. Agreement with experimental field topology	Between 0–2 μT depending on body location–see Fig 4.
**4** 165 MHz Upper Extremity	[50]	Maximum Temperature Increase after 139 seconds	6.0°C	6.6°C measured 8°C simulated–see Fig 5

Though not shown below, the electromagnetic simulation produced surface currents along the metallic implant in excess of 14 A/m current amplitude. As anticipated, the simulated *SAR* and temperature had high values along the end tips of the implant.

### Example 3: $B_{1}^{+}$ maps for 3T birdcage MRI whole body coil

Experimental [47,48] $B_{1}^{+}$ field maps for the problem geometry of Fig 6A are show in two planes in Fig 6B–6E. Fig 6B and 6C depict the $B_{1}^{+}$ map in an axial plane at the abdominal region of the VHP Female mode and the corresponding measured data obtained for healthy volunteer 2 [47]. The two remaining plots in Fig 6 depict the same $B_{1}^{+}$ field map in a coronal plane throughout the center of the model.

The field profiles depicted in Fig 6 are topologically similar to the measured profiles provided in [47,48]. Emphasize that the peak field values are approaching exactly 2 μT in either case when whole-body *SAR* of 0.51 W/kg is enforced. In both instances, hot spots are noted around the inner arm edges and the intensity of the field is shown to dissipate as it moves into the center of the model/patient. Peak values in the central region are slightly under 1 μT for both measured and simulated results.

Variation between the experimentally captured data and the results using the VHP Female v3.0 model are due to the fact that the body composition, arm positions, and the coil type are not entirely equivalent. However, the general pattern and the absolute peak values correspond very well between the experimental and simulated sources and demonstrate that the VHP Female v3.0 human model will generate representative field distributions under these conditions.

*SAR* values for this case have also been simulated and recorded. Though not shown here, they also match the data in [47,48] with the peak values approaching 4 W/kg in ether case, and with higher values at the outer portions of the arms.

### Example 4: Tissue heating due to single-loop coil close to skin surface at 165 MHz

Fig 7A and 7B replicate the experimental problem geometry [49]. The simulated *SAR* generated in a plane through the VHP Female forearm directly above the center of the coil is shown in Fig 7C and 7D as compared with the experiment [49]. The peak *SAR* value experienced in the outermost model layer is approximately 450 W/kg, consistent with the value reported in [49] to within 5%. There is a lower value of *SAR* within the second layer (fat tissue) and it again rises in the muscle tissue. As expected, due to their material properties, higher *SAR* values are displayed in the nerve and cardiovascular tissues.

The simulated and measured [49] thermal maps within the forearm in a plane directly above the antenna center are given in Fig 7E and 7F after 139 seconds of coil operation. The peak simulated temperature change is about 8°C, which is slightly higher than the measured 6°C change reported in [49]. It should be noted that the peak simulated value of 8 deg reported here is in almost a perfect agreement with the 8.8°C simulated temperature change reported in [49].

The spatial temperature map is also consistent with the measured map despite the different wrist geometry and tissue composition. There is some difference in the observed depth of temperature change. This is likely mostly due to the fat layer of the VHP Female model which is thicker than the subject reported in [49].

## Discussion

### Initial model validation by co-registration

Since the original VHP-Female image dataset with the isotropic resolution of 0.33 mm is freely and publicly available, the co-registration validation performed in this study is also made public [24]. It is pertinent to VHP-Female model v5.0, which has a larger number of individual muscles than the model v3.0.

### Review of subjects, settings, and results used in validation and comparison examples

During a validation study, simulated or measured data should be compared to an independent modeling or measuring technique. Ideally, when performing such a validation study, the setup of the validation should closely resemble the simulation or measurement that is being validated. As an example, in our study it would be ideal to have the same human body model in both the simulation/measurement and the validation setup. However, since the human body model that we want to validate in our study is deceased, this is not a possibility. Therefore, comparison with the results for different human body models and/or subjects is used in this study. When possible, the attempt is made to use data obtained from human body models that are comparable to the VHP-Female model.

Validation example #1 (whole body *SAR* and the maximum *SAR*_1*g*_(***r***) evaluation) is a purely modeling study at 64 MHz. The same original dataset from the US National Library of Medicine was used for human model construction in both the cases, along with the nearly identical coil dimensions and settings. As a result, an excellent agreement in whole body *SAR* and in the maximum *SAR*_1*g*_(***r***) was obtained in Table 1 despite the different model type (voxel vs CAD) and the different solver (FDTD vs FEM).

Comparison example #2 (implant heating in the ASTM phantom at 64 MHz) also used identical coil construction, dimensions, and settings, but the implant temperature was compared with that simulated within the femur of the VHP-Female model. Despite the obvious field differences and a slightly different implant position caused by human anatomy, the implant temperature dynamics (Figs 4 and 5 and Table 2) are rather similar in both cases, for this particular anatomical location and the particular patient landmark. These are particularly relevant results given that assessment of RF safety for medical implants generally consists of obtaining measurements from inside a saline gel phantom and conducting simulations using computational human body models. It is therefore critical that these body models are anatomically correct and have been validated for this purpose. Ideally, a suite of human body models, characterizing a range of age, sex, BMI, and other pertinent characteristics, is employed given that a single model may not provide enough evidence to guarantee the safety of a medical implant. Subsequent models could follow a similar validation procedure as presented here.

Validation example #3 ($B_{1}^{+}$ map at 128 MHz *in vivo*) employs a younger male subject with the closest weight and a coil of a different length (but with the same diameter and mimicking the same birdcage resonator), which results in a good agreement, both qualitative and quantitative, in the $B_{1}^{+}$ maps in the transverse plane (Fig 6B and 6C). Some disagreement in the coronal plane (Fig 6D and 6E) could likely be due to different arm positions (beside body in [47]).

Validation example #4 (*in vivo* heating of a human arm at 165 MHz by a single loop antenna) employs the identical antenna dimensions, settings and position as well as nearly the same arm width with respect to the antenna size. However, the arm cross-sections are quite different (cf. Fig 7) since the arm of the VHP-Female model is not supported while the experiment assumes some arm support. Despite this fact, a decent agreement in the measured *SAR* and especially in temperature rise is obtained (cf. Fig 7).

### Other comparison results

A comparison with a variety of other modeling reference sources [66–76] has also been performed both at 1.5 T and 3 T. It was found that the *SAR* results calculated when using the VHP Female v3.0–5.0 model are strictly within the bounds of all other reported values.

## Conclusions

This study provided a reasonably systematic validation and comparison of the VHP-Female CAD v.3.0–5.0 surface-based computational human model starting with the segmentation validation and following four different application examples. The target context of use is a non-clinical assessment model for female patients of age 50–70 years with a BMI of 30–36 during 1.5 and 3.0 T based MRI procedures.

It was found that given the same or similar coil settings, the computational human model generates meaningful results for *SAR*, $B_{1}^{+}$ field, and temperature rise when used in conjunction with the 1.5 T birdcage MRI coils or at higher frequencies corresponding to 3 T MRI.

Notably, the temperature rise deviation from experiment did not exceed 2.75° C for three different heating scenarios considered in the study. Two of them included the long nail orthopaedic implant in the phantom and in the body, respectively. The relative temperature rise difference there was 10% and 25%, respectively. The higher error for the implant in the body is to be expected. In the last example for *in vivo* heating of the human arm, the relative temperature rise difference was 20%.

Therefore, the tested human model could likely be applied to the safety evaluation of implantable medical devices within this temperature error margin.

The approach taken in this study might also be used as an example for validation of similarly developed computational human models from no longer living subjects.

## Supporting information

S1 Appendix Tuning procedure for the MRI coil used in this study. (DOCX)
