# Supplementary material for: Visible Human Project® female surface based computational phantom (Nelly) for radio-frequency safety evaluation in MRI coils
Source: PLoS One. 2021 Dec 10;16(12):e0260922. doi: 10.1371/journal.pone.0260922 (PMC8664205; doi:10.1371/journal.pone.0260922)
Supplement: S1 Appendix — (DOCX) [file pone.0260922.s001.docx]

Appendix A. Tuning RF Transmit Coil


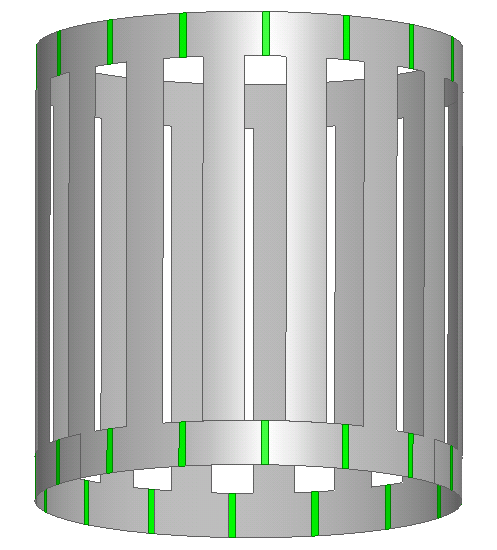


**Figure A1.** A generic high-pass birdcage MRI RF coil used in simulations. Tuning capacitors and/or driving ports are marked green.

As a base RF transmit test coil, we will consider here the simple generic high-pass birdcage coil shown in Figure A1. The coil model may have a variable number of rungs; the model shown has 16 rungs and 32 matching capacitors; it is fully parameterized. Every matching capacitor may also represent a port as described below. The coil can be scaled to be used either for the head or as a full-body coil of a varying size. Other topologies may be handled similarly.

The entire problem is quite complex since RF coil designs vary widely. Furthermore, RF coils need to be tuned and their ports need to be matched. The foundations of RF coil tuning and matching are given in Refs. [1]-[8].

**Figure A2.** VHP-Female model v.3.0 BASE in another full-body high-pass birdcage MRI RF coil.

An accurate tuning and matching procedure requires two distinct software packages: a full-wave CEM simulator coupled with a circuit co-simulator for multiport circuit modeling (with up to 64 ports for modern RF coils).

We will use an approach, which does not require the dedicated circuit co-simulator. The idea consists of the design and usage of an “ideal” coil model, which is already “perfectly” tuned and matched at all ports. Such a concept will be described and justified below. Our method will be subdivided into steps. The corresponding MATLAB script is given at the end of the Appendix.

*Step 1. Coil resonances.* An RF MRI coil such as the most common high-pass birdcage coil shown in Figure A1 above and in Figure A2 is a familiar LC resonator. The inductance $L$ (“mass”) is due to vertical coil conductors (rungs) seen in Figures A1 and A2, while the capacitance $C$ (inverse “stiffness”) is due to the lumped tuning capacitors marked green in Figures A1 and A2. The number of distinct coil resonances is equal to the number of the tuning capacitors (“springs”) in Figures A1 and A2. The resonances appear in pairs exactly corresponding to the pairs of upper and lower capacitors in Figures A1 and A2, respectively. Magnetic fields of each pair of the resonant modes are perpendicular to each other. When combined, these two perpendicular fields make it possible to create a circular polarization.

*Step 2. Finding all coil resonances.* In order to find all resonances of the coil and then select the main resonant mode pair, we will consider the coil in Figures A1 and A2 as a generic N-port linear network shown in Figure A3. Every tuning capacitance $C_{0}$ (with an impedance of $Z_{0}$ at the a priori known resonant frequency) is now becoming an independent port driven by an RF generator. The N-port network is routinely modeled in any commercial CEM software at the known resonant frequency. As a result, an impedance matrix $\hat{\boldsymbol{Z}}$ is obtained, which relates the port voltages $\vec{\boldsymbol{V}}$ and currents $\vec{\boldsymbol{I}}$ by:

$\left[ \begin{matrix} V_{1} \\ \begin{matrix} V_{2} \\ \ldots\\ V_{N} \end{matrix} \end{matrix} \right]\boldsymbol{=}\left[ \begin{matrix} \begin{matrix} Z_{11} & Z_{12} \\ Z_{21} & Z_{22} \end{matrix} & \begin{matrix} \boldsymbol{\ldots} & Z_{1N} \\ \boldsymbol{\ldots} & Z_{2N} \end{matrix} \\ \begin{matrix} \boldsymbol{\ldots} & \boldsymbol{\ldots} \\ Z_{N1} & Z_{N2} \end{matrix} & \begin{matrix} \boldsymbol{\ldots} & \boldsymbol{\ldots} \\ \boldsymbol{\ldots} & Z_{NN} \end{matrix} \end{matrix} \right]\boldsymbol{\times}\left[ \begin{matrix} I_{1} \\ \begin{matrix} I_{2} \\ \ldots\\ I_{N} \end{matrix} \end{matrix} \right]$ (1)

Mathematically, the *N* distinct coil resonances correspond to N eigenvalues and N eigenvectors of the impedance matrix $\hat{\boldsymbol{Z}}$, very similar to the eigenvalues of the stiffness matrix in mechanical engineering of lumped resonant systems. Any eigenvalue is equal to $Z_{0}$, i.e., to the port impedance which is the same for all ports, i.e., $\vec{\boldsymbol{V}}\boldsymbol{=}Z_{\boldsymbol{0}}\vec{\boldsymbol{I}}$ . From Eq. (1), the corresponding eigenvalue problem is obtained in the form:

$\left[ \begin{matrix} \begin{matrix} Z_{11} & Z_{12} \\ Z_{21} & Z_{22} \end{matrix} & \begin{matrix} \boldsymbol{\ldots} & Z_{1N} \\ \boldsymbol{\ldots} & Z_{2N} \end{matrix} \\ \begin{matrix} \boldsymbol{\ldots} & \boldsymbol{\ldots} \\ Z_{N1} & Z_{N2} \end{matrix} & \begin{matrix} \boldsymbol{\ldots} & \boldsymbol{\ldots} \\ \boldsymbol{\ldots} & Z_{NN} \end{matrix} \end{matrix} \right]\boldsymbol{\times}\left[ \begin{matrix} I_{1} \\ \begin{matrix} I_{2} \\ \ldots\\ I_{N} \end{matrix} \end{matrix} \right]\boldsymbol{=}Z_{0}\left[ \begin{matrix} I_{1} \\ \begin{matrix} I_{2} \\ \ldots\\ I_{N} \end{matrix} \end{matrix} \right]$ (2)

We will solve Eq. (2) using MATLAB or another generic engineering software package. As a result, we will obtain *N* resonances: *N* distinct values of port impedance $Z_{0}$ and *N* distinct values of vector $\vec{\boldsymbol{I}}$ (electric currents at the ports).

*Step 3. Finding two dominant coil resonances.* The pair of desired resonant modes must generate a magnetic field that is as homogeneous as possible within the coil and that is directed along one of the horizontal axes in Figures A1 and A2 (either along the *x*-axis or along the *y*-axis). In this case, the current distribution $\vec{\boldsymbol{I}}$ for every mode in the pair must be a sinusoidal function of the polar angle $\varphi$ around the coil with exactly one period. The corresponding mode strength, $m$, is therefore given by a Fourier coefficient,

**Figure A3.** RF MRI coil terminated into *N* ports (with impedances $Z_{0}$).

$m=abs\left( \sum_{n=1}^{N/2} exp(4\pi jn/N)I_{n} \right)$ (3)

The analysis of mode strength from Eq. (3) will show two nearly equal clear peaks (10-50 times higher than the rest of the values) corresponding to the two desired modes. Yet another approach is to realize that the main resonance is exactly at the upper end of the group of coil resonances that lead to positive values of $C_{0}$ (with the two largest values of $C_{0}$).

*Step 4. Constructing a perfectly matched coil with 1 W total input power.* In practice, only a limited number of ports are driven (typically two or four). In order to obtain a solution exactly corresponding to the two resonant modes, we will drive all ports and assume ideal matching to the characteristic impedance $Z_{c}$ (typically $Z_{c}=50 \Omega$). Given the incident normalized power wave at the ports as $\vec{\boldsymbol{A}}=\vec{\boldsymbol{V}}\boldsymbol{+}Z_{c}\vec{\boldsymbol{I}}$ and the reflected power wave as $\vec{\boldsymbol{B}}=\hat{\boldsymbol{S}}\vec{\boldsymbol{A}}$, where $\hat{\boldsymbol{S}}$ is the known scattering matrix (expressed through the impedance matrix and vice versa), we find the total input power per mode $i=1,2$ as

$P_{i}=1/2\left( \left| \vec{\boldsymbol{A}} \right|\cdot\left| \vec{\boldsymbol{A}} \right|-\left| \vec{\boldsymbol{B}} \right|\cdot\left| \vec{\boldsymbol{B}} \right| \right)$ (4)

Then, we normalize every main mode as $\vec{\boldsymbol{A}}=\vec{\boldsymbol{A}}\boldsymbol{/}\sqrt{2P_{i}}$. Finally, a right-handed circularly-polarized magnetic field $\boldsymbol{B}_{1}^{+}$ with 1 W total input power will be generated by the port sources ${\vec{\boldsymbol{S}}=\vec{\boldsymbol{A}}}_{1}+j{\vec{\boldsymbol{A}}}_{2}$, which should be directly substituted into the CEM software package. This provides a concise description of our model.

*Step 5. Testing the tuning method: method accuracy within unloaded coil*. We will consider an unloaded (without the body) coil shown in Figure A1 with 16 rungs, 32 matching capacitors (each of which can be connected to a driving port), with the diameter of 604 mm and the length of 650 mm. All relevant simulations are performed at 63.87 MHz ($B_{0}=1.5 T$) using ANSYS Electronics Desktop (ANSYS HFSS) v.16.2, Perfectly Matched Layer (PML) termination, and 12 adaptive mesh refinement passes.

A comparison will be made with an accurate practical solution [1], which uses the circuit co-simulator to drive the coil with two ports spaced by 90° (ports 1 and 5) and also establishes the port-matching networks. This solution reports the value of $C_{0}=106.2 \mathrm{pF}$, while our method reports $C_{0}=106.23 \mathrm{pF}$ for one resonant mode and $C_{0}=106.16 \mathrm{pF}$ for another mode. The agreement is almost exact! The corresponding distributions of the magnitude of magnetic field $\boldsymbol{B}_{1}^{+}$ within the shielded coil at the coil center are shown in Figure A4b, c given the same input power of 1 W and the same color scale. Both solutions are very close to each other, although our solution generates slightly more symmetric and homogeneous field, since all ports are now driven as appropriate.

**Figure A4.** Magnetic field distribution within the unloaded RF coil at 1 W input power. a) – Coil geometry; b) – practical solution with two matched driving ports; c) – our ideal model with all matched driving ports. The same color scale is used on both figures.

*Step 6. Testing the tuning method: accuracy within the loaded coil*. We will apply our method to compute the circularly-polarized magnetic field $\boldsymbol{B}_{1}^{+}$ in an ideally matched/tuned coil loaded with the VHP-Female model. Figure A5 shows simulations with the VHP-Female model for the magnitude distributions of the magnetic field $\boldsymbol{B}_{1}^{+}$ within the shielded coil in a transverse plane passing through the coil center. The same parameters as in Figure A4 are used but the color scale is different. All relevant simulations are performed using ANSYS Electronics Desktop (ANSYS HFSS) v.16.2, PML terminations, and 3 adaptive mesh refinement passes.

We will consider the loaded (with the body) coil shown in Figure A1 with 16 rungs, 32 matching capacitors (each of which can be connected to a driving port), with the diameter of 640 mm and the length of 689 mm at the head landmark (the center of the coil coincides with the top of the cerebellum).

A comparison is again made with the accurate practical solution [1], which uses the circuit co-simulator to drive the coil with two ports spaced by 90° (ports 1 and 5) and also establishes the port-matching networks.

Although this is a rather complicated case for coil tuning since the coil is loaded highly asymmetrically, both solutions again predict quite similar magnetic field magnitudes. Our generic solution generates a slightly more symmetric and homogeneous field.

*Step 7.* $SAR$ *computations at the head landmark*. We will apply our method to compute $\mathrm{SAR}$ in an ideally matched/tuned coil loaded with the VHP-Female model. Figure A6 shows simulations for the distributions of the local non-averaged $\mathrm{SAR}$ within the shielded coil at the coil center for the VHP-Female model. The same coil, parameters and the landmark as in Figure A5 are used. All relevant simulations are performed using ANSYS Electronics Desktop (ANSYS HFSS) v.16.2, PML terminations, and 3 adaptive mesh refinement passes.

**Figure A6.** SAR distribution in the cranium within the RF coil loaded with the VHP-Female v.3.0 model at 1 W input power. a) – Practical solution with two matched driving ports; b) – our ideal model with all matched driving ports. The same color scale is used on both figures. Shown are the SAR values in the cortical transverse plane, the fields in the cerebellum object and in the “average body” object.

**Figure A5.** Magnetic field distribution within the RF coil loaded with the VHP-Female v.3.0 model at 1 W input power. a) – Landmark of the model inside the birdcage; b) – practical solution with two matched driving ports; c) – our ideal model with all matched driving ports. The same color scale is used on both figures. Shown are the fields in the coil volume, the fields in the cerebellum object and in the “average body” object.

A comparison is again made with the accurate practical solution [1], which uses the circuit-co-simulator to drive the coil with two ports spaced by 90° (ports 1 and 5) and establishes the port-matching networks.

Both solutions generate similar $SAR$ distributions. At the same time, our generic solution shown in Figure A6b generates a smoother and likely more realistic $\mathrm{SAR}$ distribution specifically near the right eye. The corresponding local $\mathrm{SAR}$ maximum may be caused by an insufficient quality of the local FEM mesh or by other reasons.

**References**

1. Lemdiasov R, Obi A, Ludwig R. A Numerical Postprocessing Procedure for Analyzing Radio Frequency MRI Coils. Concepts in Magnetic Resonance Part A. 2011; 38A(4): 133–147.
2. Kozlov M, Turner R. Fast MRI coil analysis based on 3-D electromagnetic and RF circuit co-simulation. J Magn Reson. 2009; 200: 147–152.
3. Kozlov M, Turner R. A comparison of Ansoft HFSS and CST Microwave Studio simulation software for multi-channel coil design and SAR estimation at 7 T MRI. PIERS Online. 2010; 4: 395–399.
4. Roemer PB, Edelstein WA, Hayes CE, Souza SP, Mueller OM. The NMR phased array. Magn Reson Med. 1990; 16: 192–225.
5. Chen J, Feng Z, Jin J-M. Numerical simulation of SAR and B1-field inhomogeneity of shielded RF coils loaded with the human head. IEEE Trans Biomed Eng. 1998; 45: 650–659.
6. Jiao D, Jin J-M. Fast frequency-sweep analysis of RF coils for MRI. IEEE Trans Biomed Eng. 1999; 46: 1387–1390.
7. Reza S, Vijayakumar S, Limkeman M, Huang F, Saylor C. SAR simulation and the effect of mode coupling in a birdcage resonator. Conc Magn Reson Part B: Magn Reson Eng. 2007; 31: 133–139.
8. Ibrahim TS, Lee R, Baertlein BA, Abduljalil AM, Zhu H, Robitaille P-ML. Effect of RF coil excitation on field inhomogeneity at ultra high fields: a field optimized TEM resonator. Magn Reson Imaging. 2001; 19: 1339–1347.
9. Oh S, Webb AG, Neuberger T, Park B, Collins CM. Experimental and numerical assessment of MRI-induced temperature change and SAR distributions in phantoms and in vivo. Magn Reson Med. January 2010; 63(1): 218-23. PMCID: PMC2836721.
10. Homann H. SAR Prediction and SAR Management for Parallel Transmit MRI. Karlsruhe Translations on Biomedical Eng. 2012; 16: 1-124.
11. Collins CM, Li S, Smith MB. SAR and B1 Field Distribution in a Heterogeneous Human Head Model with a Birdcage Coil. Magnetic Resonance in Medicine. 2005; 40(6): 847-856.
12. Rennings A, Chen L, Otto S, Erni D. B1-Field Inhomogeneity Problem of MRI: Basic Investigation on a Head- Tissue-Simulating Cylinder Phantom Excited by a Birdcage-Mode. 42nd European Microwave Conference, The Netherlands, Amsterdam, 2012 November 1; pp. 542-545.
13. Tomas BP, Li H, Anjum MR. Design and Simulation of a Birdcage Coil using CST Studio Suite for Application at 7T. IOP Conference Series: Materials Science and Engineering. 2013 February 1; 51(1):1-6.
14. Yeo DTB, Wang Z, Loew W, Volgel MW, Hancu I. Local SAR in High Pass Birdcage and TEM Body Coils for Multiple Human Body Models in Clinical Landmark Positions at 3T. Journal of Magnetic Resonance Imaging. 2011 May; 33(5): 1209 – 1217. PMCID: PMC3081105.
15. Liu W, Collins CM, Smith MB. Calculation of B1 Distribution, SAR, and SNR for a Body-size Birdcage Coil Loaded with Different Human Subjects at 64 and 128 MHz. Applied Magnetic Resonance, 2005 March; 29(1): 5-18. PMID:23565039 PMCID: PMC3615460.
16. Cabot E, Christ A, Kuster N. Whole Body and Local SAR in Anatomical Phantoms Exposed to RF Fields from Birdcage Coils. Proceedings of the 29^th^ General Assembly of the International Union of Radio Science. 2008 August 7-16. Chicago, USA.;
17. Kozlov M, Turner R. RF Transmit Performance Comparison for Several MRI Head Arrays at 300 MHz. Progress in Electromagnetic Research Symposium Proceedings. 2013 March 28 Taipei: 1052-1056.
18. Kozlov M, Bazin PL, Möller HE, Weiskopf N. Influence of Cerebrospinal Fluid on Specific Absorption Rate Generated by 300 MHz MRI Transmit Array. 10th European Conference on Antennas and Propagation (EuCAP). 2016 April. Davos. Switzerland. pp. 1-5.
19. Taflove A. *Computational Electrodynamics, The Finite Difference Time Domain Approach*, Third Ed., Artech House, Norwood, MA, 2005.
20. Thoma P, Weiland, T. A consistent subgridding scheme for the finite difference time domain method," Int. J. of Numerical Modelling, vol. 9, pp. 359-374, 1996.

**MATLAB Tuning Script: Computing sources (or excitations) based on the port data obtained from CEM simulations**

% Eigenmode method for coil tuning

clear all

%% Import port matrices

Z_matrix;

S_matrix;

Z = squeeze(Z);

S = squeeze(S);

N_rungs = size(Z,1)/2;

%% Construct eigenvectors/eigenvalues of the Z-matrix

Z = -Z; % negative impedance matrix

[I, D] = eig(Z); % compute eigenmodes

lambda = diag(D);

C0 = real(1./(1j*(2*pi*f)*lambda)); %terminating capacitances for each mode

keep = C0 > 0; % filter out negative capacitances

C0 = C0(keep);

I = I(:,keep);

%% Find mode signature and select the dominant resonant modes

mode_signature = exp(2j*pi*(0:N_rungs-1)/N_rungs); % sinusoidal mode signature

mode_strength = abs(mode_signature * I(1:N_rungs,:));

[~, ind] = sort(mode_strength, 'descend'); % keep the two modes with the largest mode strength

C0 = C0(ind(1:2));

I = I(:,ind(1:2));

C0_pF = C0 * 1e12

%% Find excitation sources

Z0_S = 50;

V = Z * I; % voltages for the coil ports

A = V + Z0_S * I; % incident normalized power waves

B = S * A; % reflected normalized power waves

P_raw = 0.5 * (sum(abs(A).^2) - sum(abs(B).^2)); % power dissipated in the coil

A(:,1) = A(:,1) / sqrt(2 * P_raw(1)); % normalize coil drive power to 1W (0.5W per mode)

A(:,2) = A(:,2) / sqrt(2 * P_raw(2));

sources = A * [1, 1; 1j, -1j]; % combine modes to create circular polarization

mode_strength = abs(mode_signature * sources(1:N_rungs,:));

[~, ind] = sort(mode_strength); % sort sources by circular polarization direction

sources = sources(:, ind);

%% Output excitation sources in EXCEL format

f = fopen('sources1_eigZ.csv','wt');

fprintf(f, 'Source,Magnitude,Phase');

for i=1:size(sources,1)

fprintf(f, '\n%u:1,%.8gW,%.8gdeg', i, 0.5 * abs(sources(i,1)).^2, angle(sources(i,1)) * 180 / pi);

end

fclose(f);

f = fopen('sources2_eigZ.csv','wt');

fprintf(f, 'Source,Magnitude,Phase');

for i=1:size(sources,1)

fprintf(f, '\n%u:1,%.8gW,%.8gdeg', i, 0.5 * abs(sources(i,2)).^2, angle(sources(i,2)) * 180 / pi);

end

fclose(f);
